# Enhanced autophagic retrograde axonal transport by dynein intermediate chain upregulation improves Aβ clearance and cognitive function in APP/PS1 double transgenic mice

**DOI:** 10.18632/aging.103382

**Published:** 2020-06-24

**Authors:** Fanlin Zhou, Xiaomin Xiong, Shijie Li, Jie Liang, Xiong Zhang, Mingyuan Tian, Xiaoju Li, Minna Gao, Li Tang, Yu Li

**Affiliations:** 1 Institute of Neuroscience, School of Basic Medicine, Chongqing Medical University, Chongqing 400016, China; 2 Key Laboratory for Biorheological Science and Technology of Ministry of Education, Chongqing University, Chongqing University Cancer Hospital, Chongqing 400044, China; 3 Chongqing Key Laboratory of Translational Research for Cancer Metastasis and Individualized Treatment, Chongqing University Cancer Hospital, Chongqing 400030, China; 4 Department of Endocrinology, The Second Affiliated Hospital, Chongqing Medical University, Chongqing 400016, China; 5 Department of Pathophysiology, Chongqing Medical University, Chongqing 400016, China

**Keywords:** Alzheimer’s disease, dynein intermediate chain, autophagy, axonal transport

## Abstract

Autophagosome accumulation is observed in the distal axons of Alzheimer disease (AD) patients and AD animal models, suggesting that deficient retrograde transport and impaired autophagic clearance of beta-amyloid (A β) contribute to AD pathogenesis. Expression of the retrograde axonal transport-related protein dynein intermediate chain (DIC) is also reduced in AD patients, but the contributions of DIC to AD pathology remain elusive. This study investigated the effects of DIC expression levels on cognitive function, autophagosome axonal transport, and A β clearance in the APP/PS1 double transgenic mouse model of AD. Autophagic activity was enhanced in the hippocampus of young (3-month-old) AD mice, as evidenced by greater expression of autophagosome markers, lysosome markers, axonal transport motors (including DIC), and dynein regulatory proteins. The expression levels of autophagosome markers remained elevated, whereas those of autophagic and axonal transport proteins decreased progressively with age, accompanied by spatial learning and memory deficits, axonal autophagosome accumulation, and A β deposition. Knockdown of DIC exacerbated while overexpression improved axonal transport, autophagosome maturation, Aβ clearance, and spatial learning and memory in aged AD mice. Our study provides evidence that age-dependent failure of axonal autophagic flux contributes to AD-associated neuropathology and cognitive deficits, suggesting DIC as a potential therapeutic target for AD.

## INTRODUCTION

Macroautophagy, or simply autophagy, maintains cell viability, particularly under stress, by recycling the constituent components of damaged macromolecules and organelles through a specific autophagic vesicle–lysosome degradation pathway [1–4]. In neurons, nascent autophagic vesicles (autophagosomes) are mainly formed in distal axons and thus require long-distance retrograde transport to merge with degradative lysosomes located near the cell body for efficient recycling [5–7]. This dynamic process, termed autophagic flux, is essential to prevent the accumulation of damaged molecules and organelles with age [8].

Projection neurons exhibit high rates of anterograde and retrograde axonal transport for maintenance of synaptic function and retrograde signaling [7]. The molecular motor kinesin mediates autophagosome anterograde transport, whereas the molecular motor dynein mediates retrograde transport, and these two molecular motors are co-localized at neuronal autophagosomes [9]. Newborn autophagosomes move toward and away from the cell body along the axonal microtubules [9] but show predominantly one-way retrograde movement near the cell body [5, 6, 10]. The regulation of these molecular motors, particularly dynein, is essential for this transition from bidirectional to highly unidirectional retrograde movement. For retrograde autophagosome transport, dynein forms a complex with dynactin (dynamic actin), which is expressed in two main subunit forms: P150 (P150Glued) and P50 (Dynacin2). Other adaptor proteins selectively promote or inhibit autophagosome transport and autophagosome-lysosome maturation through direct binding to kinesin and dynein [11]. Of these proteins, Rab7 is a vesicular transport-related protein that promotes the fusion of autophagosomes and lysosomes by regulating their movement on the microtubules [12, 13]. Rab7 binds to oxysterol-binding protein-related protein 1L (ORP1L), and then connects to dynein–dynactin [13]. Newborn autophagosomes in distal axons must associate with lysosome-associated membrane protein-2 (LAMP2) and Rab7 for subsequent binding to late lysosome-related motor proteins required for long-distance retrograde transport [14, 15]. The main function of autophagy under stress is to protect cells by degrading harmful substances. Under prolonged or severe stress, however, autophagic capacity may be exceeded or specific autophagic processes dysregulated, resulting in accumulation of degraded proteins and damaged organelles in the axons. Many of these accumulated molecules are cytotoxic, such as the Alzheimer disease (AD)-associated protein beta-amyloid (Aβ) [16, 17].

The main pathological hallmarks of AD are the formation of intracellular neurofibrillary tangles (NFTs) and extracellular senile plaques containing Aβ [18–20]. As Aβ is neurotoxic, enhanced clearance is considered central to prevention and treatment of AD [21, 22]. It is speculated that deficient macroautophagy may underlie Aβ accumulation, as the distal axons of patients with AD exhibit large numbers of immature autophagosomes. In the early stages of the disease, autophagy is activated, accelerating the clearance of Aβ and damaged organelles, thereby preventing disease development [23]. However, with disease progression, autophagosomes accumulate and Aβ and damaged organelles are not degraded successfully, which in turn can induce autophagic stress, organelle damage, and ultimately cell death [23–25]. Therefore, balanced autophagosome production and degradation are a prerequisite for neuroprotection in AD. In our previous study, we observed that several autophagosomes could not successfully combine with lysosomes to form mature autophagic lysosomes in AD model N2a/APP695swe cells transfected with a double-labeled autophagic adenovirus (mRFP-GFP-LC3) [26]. Dynein intermediate chain (DIC) is required for autophagosome transport and autophagic lysosome maturation, and it has been reported that DIC expression is downregulated in the brain of patients with AD [27]. However, the specific mechanisms mediating autophagosome retrograde transport dysfunction in AD and the specific contributions of DIC remain unclear.

In this study, we examined the dynamics of axon transport and the associations with autophagic Aβ clearance and cognitive function in APP/PS1 double transgenic AD model mice. We first compared the spatial learning and memory performance of AD mice to wild-type (WT) littermates during aging and examined the changes in autophagy and axonal transport-related protein expression in hippocampus. Further, we examined the effects of hippocampal DIC overexpression or knockdown on lysosomal function, axonal transport-related protein expression, autophagosome and insoluble Aβ aggregation, and spatial learning and memory.

## RESULTS

### Cognitive function was impaired and insoluble Aβ deposition was increased in hippocampus of APP/PS1 double transgenic mice

**Figure 1:**
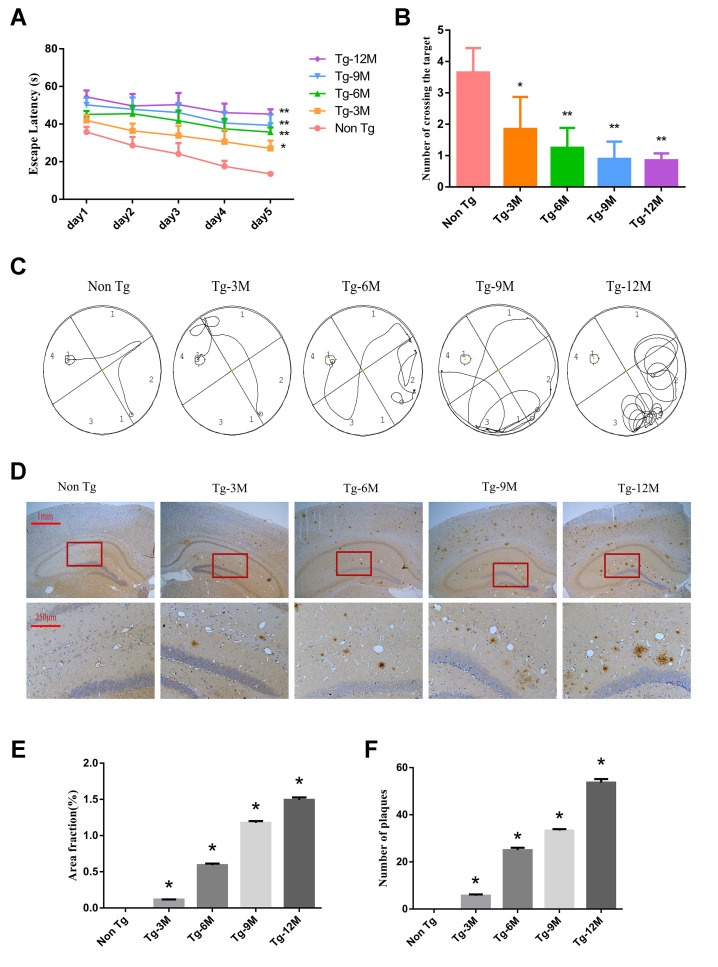
**Cognitive function was impaired and insoluble Aβ deposition increased in hippocampus of APP/PS1 double transgenic mice.** (**A**) The escape latency during 5 days of the MWM test. (**B**) Number of crossing the target in the last day of the MWM test. (n=5, two-way repeated-measures ANOVA, Tukey's test, * P<0.05,** P<0.01, versus Non-Tg) (**C**) The representative route track map of each group of mice. (**D**) Immunohistochemical staining shows amyloid protein expression in the hippocampus of different groups (Scale bar=1 mm, Scale bar=250 μm). (**E**, **F**) Area fraction and number of plaques of amyloid protein in each group. (* P<0.05, versus Non Tg).

The Morris water maze (MWM) is widely used to assess hippocampus-dependent spatial learning and memory. In the MWM task, a test mouse or rat must find a hidden (submerged) escape platform in a circular pool filled with opaque water by using the surrounding visual landmarks [28]. During the learning phase (Figure 1A), 3-month-old AD model transgenic (Tg-3M) mice demonstrated significantly longer times to find the escape platform (longer escape latencies) compared to age-matched WT littermates (Non-Tg mice) (P < 0.05). Furthermore, average escape latency increased significantly with age in the Tg group (P < 0.01, P < 0.01). In the probe test for spatial memory retention (Figure 1B), Tg-3M mice made significantly fewer crossings of the former platform location than Non-Tg mice and the platform crossing frequency decreased with age (all P< 0.001). Representative swim paths are shown in Figure 1C.

These spatial learning deficits were paralleled by β-amyloid 17-24 (Aβ) plaque deposition in the hippocampus of Tg-3M mice. These plaques were large, mostly round and radial, with a deep core, and gradually increased in number with Tg mouse age (Figure 1D–1F).

### Autophagosome was accumulated and autophagy was induced in hippocampus of APP/PS1 double transgenic mice

**Figure 2:**
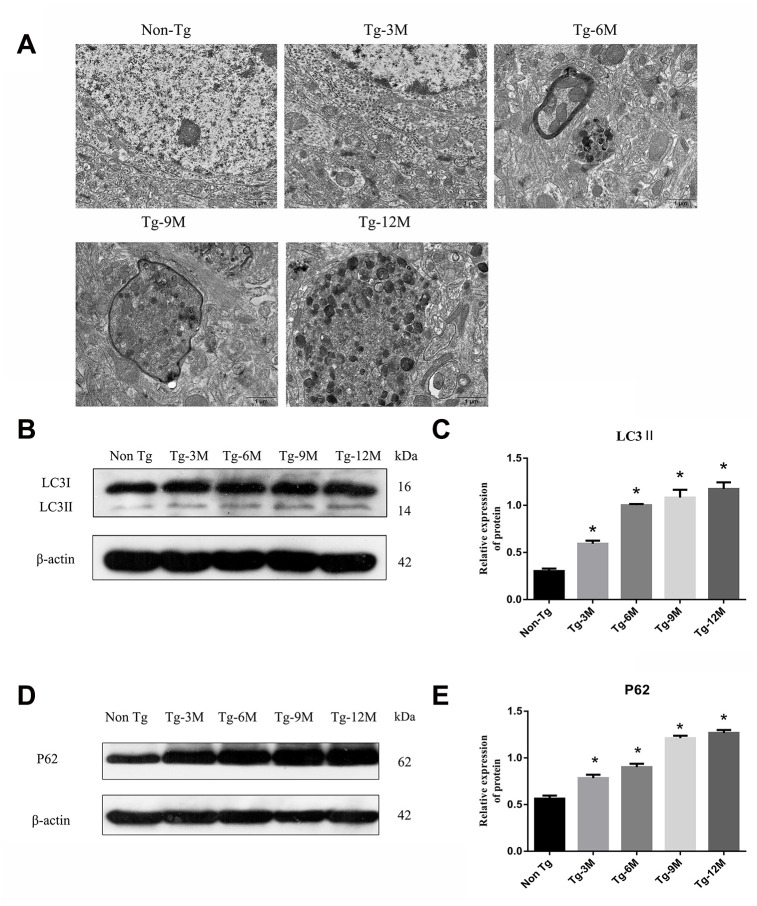
**The function of lysosome was impaired and the expression of axonal transport molecular motor was decreased in hippocampus of APP/PS1 double transgenic mice.** (**A**) Transmission electron microscopy images of each group. (**B**–**E**) Western blot analysis of (**B**, **C**) LC3II, and (**D**, **E**) P62 in each group. The data represent as mean {plus minus} SEM of a typical series of 3 experiments. (* P<0.05, versus Non Tg).

Transmission electron microscopy (TEM) revealed no autophagic structures in Non-Tg or Tg-3M hippocampi, but autophagosome number appeared to increase with age in the hippocampus of Tg mice, mainly accumulating in the axons and surrounded by microtubule-like structures (Figure 2A). Microtubule-associated protein 1 light chain 3 beta 2 (LC3II) is a phosphatidylethanolamine-conjugated autophagosome membrane protein and P62 (sequestosome 1) is a substrate of autophagic degradation used extensively as markers to monitor the progression of autophagy [8, 29]. As shown in Figure 2B and 2C, LC3II immunoexpression was significantly higher in Tg-3M mice than in Non-Tg mice, suggesting autophagic activation in the early stage of AD (P < 0.001). Moreover, LC3II expression remained higher in 6-month-old Tg mice (Tg-6M), Tg-9M mice, and Tg-12M mice compared to Non-Tg mice (all P < 0.001). In addition, consistent with autophagic activation, P62 expression was significantly higher in Tg-3M mice than in Non-Tg mice (P < 0.001) (Figure 2D and 2E) and remained higher in Tg-6M, Tg-9M, and Tg-12M mice than in Non-Tg mice (all P < 0.001).

### The function of lysosome was impaired and the expression of axonal transport molecular motor was decreased in hippocampus of APP/PS1 double transgenic mice

**Figure 3:**
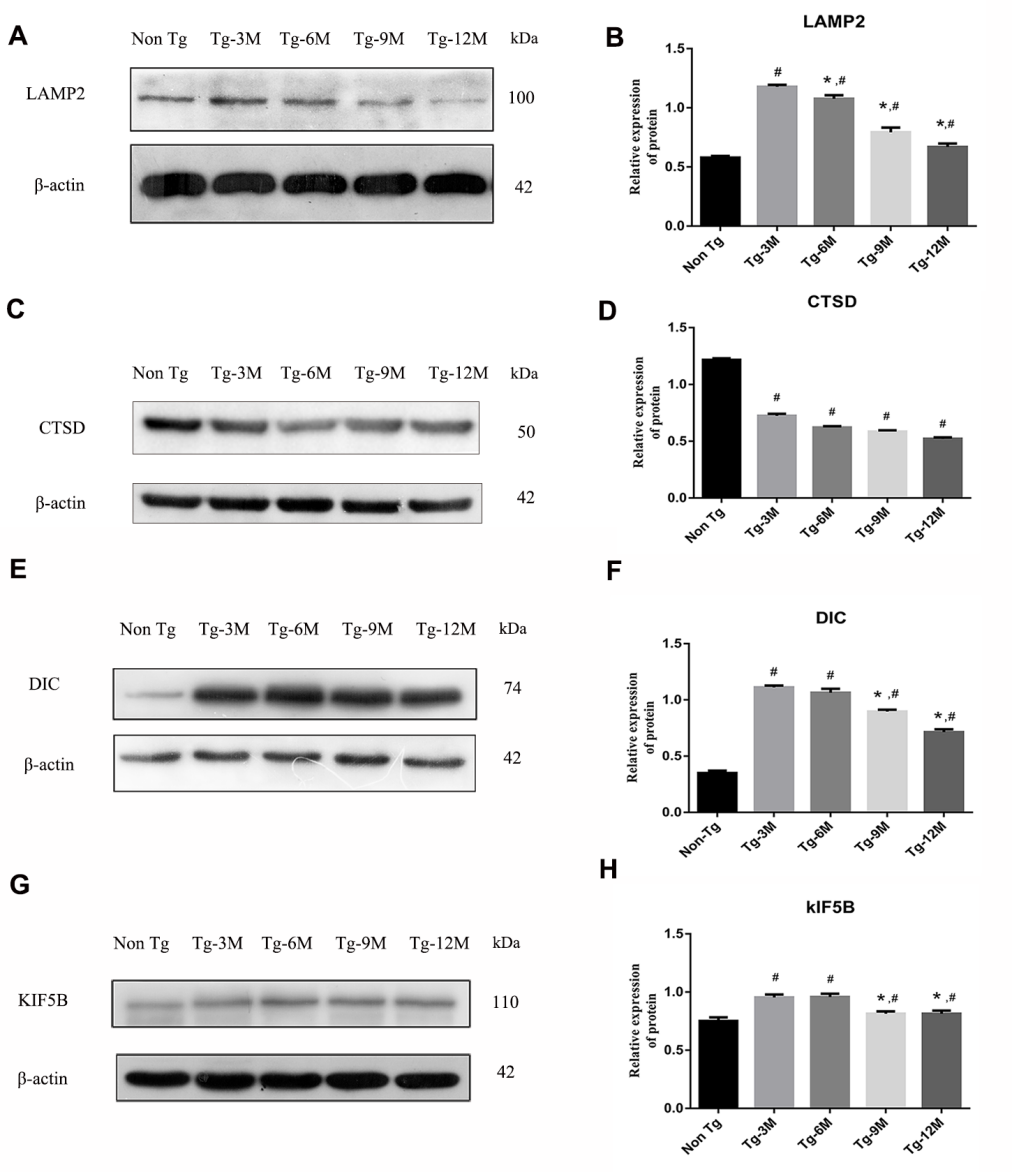
**NEW - The function of lysosome in hippocampus of APP/PS1 double transgenic mice was impaired and the expression of axonal transport molecular motor was decreased.** (**A**–**D**) Western blot analysis of LAMP2 (**A**, **B**) and CTSD (**C**, **D**) in each group. (**E**–**H**) Western blot analysis of retrograde axonal transport molecular motor DIC (**E**, **F**) and forward axonal transport molecular motor KIF 5B (**G**–**H**) in each group. The data represent as mean ± SEM of a typical series of 3 experiments. (# P<0.01, versus Non Tg; * P<0.01, versus Tg-3M).

The maturation of autophagosomes is a critical step in autophagy as damaged organelles and macromolecules can be efficiently degraded and recycled only when mature autophagosomes combine with lysosomes to form autophagic lysosomes. To detect lysosomal function in Tg and Non-Tg mice, we measured the expression of the lysosome marker proteins LAMP2 and cathepsin D (CTSD) by Western blotting (Figure 3A–3D). The expression of LAMP2 was higher in Tg-3M mice than in Non-Tg mice (P < 0.001), consistent with autophagic activation demonstrated by greater LC3II expression. However, LAMP2 expression in Tg mice decreased progressively with age (Tg-3M vs Tg-6M, Tg-9M, and Tg-12M, all P < 0.001). Similarly, the expression of CTSD was significantly higher in 3-month-old Tg mice than in Non-Tg mice, and decreased significantly with age (Tg-3M vs Tg-6M, Tg-9M, and Tg-12M, all P<0.001) (Figure 8E).

The maturation of autophagosomes depends on the axonal transport from distal axons to the cell body along microtubules and subsequent fusion with lysosomes. Therefore, the molecular motors dynein and kinesin, which drive retrograde and anterograde axonal transport, respectively, are critical for autophagosome maturation. The expression of both DIC and kinesin family member 5B (KIF5B) (Figure 3E–3H) was elevated in Tg-3M and Tg-6M mice compared to that in Non-Tg mice (all P < 0.000). However, the expression of DIC and KIF5B were reduced in Tg-9M and Tg-12M mice compared to that in Tg-3M mice (both P < 0.001).

### The expression of retrograde axon transport-related proteins P150, P50, Rab7 and ORP1L was decreased in hippocampus of APP/PS1 double transgenic mice with age

Autophagosome retrograde transport also requires multiple accessory proteins in addition to DIC, including the microtubule-associated dynactin proteins (P150 and P50) and the dynein regulators Rab7 and ORP1L. Western blotting revealed that the expression of P150 and P50 was significantly higher in Tg-3M and Tg-6M mice than in Non-Tg mice (all P < 0.001), but the expression gradually decreased with age (Tg-6M vs Tg-9M and Tg-12M, all P < 0.001). Similarly, the expression levels of Rab7 and ORP1L were significantly greater in Tg-3M and Tg-6M mice than in Non-Tg mice (all P < 0.005), and again the expression was reduced in Tg-9M and Tg-12M mice compared to that in Tg-6M mice (all P < 0.001).

### DIC improved cognitive function and reduced insoluble Aβ deposition in hippocampus of APP/PS1 double transgenic mice

To assess the effects of DIC expression levels on autophagic flux, Aβ clearance, and hippocampus-dependent cognitive function, the expression levels were manipulated using targeted siRNA and exogenous overexpression vectors. The most effective siRNA sequence was selected from three candidates using N2a cells (Figure 5A–5C). These cells were also used to test an adeno-associated virus vector. Then, DIC interference or overexpression vector was injected into the hippocampus of Tg mice, and the success of interference or overexpression was verified by western blotting (Figure 5D–5F).

**Figure 4:**
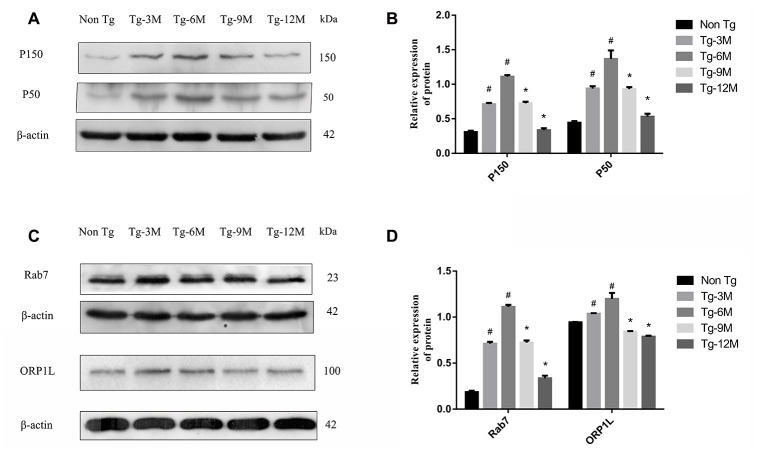
**Decreased expression of retrograde axon transport related proteins P150, P50, Rab7 and ORP1L in hippocampus of APP/PS1 double transgenic mice with age.** (**A**, **B**) Western blot analysis of dynactin proteins P150 and P50 in each group. (**C**, **D**) Western blot analysis of Rab7 and ORP1L in each group. The data represent as mean {plus minus} SEM of a typical series of 3 experiments. (# P<0.01, versus Non-Tg; * P<0.01, versus Tg-6M)

**Figure 5:**
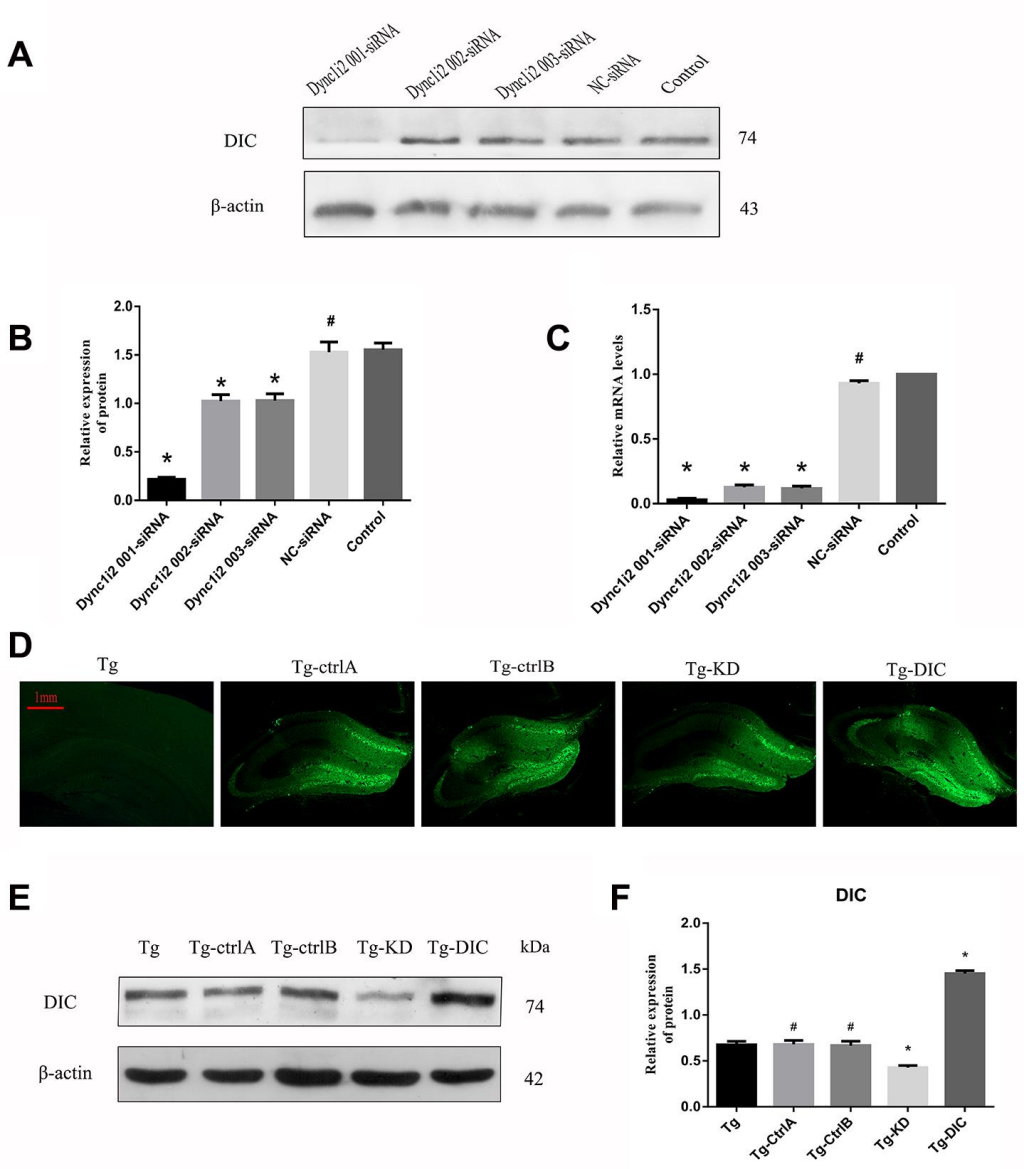
**Knockdown and overexpression of DIC in hippocampus of APP/PS1 double transgenic mice.** (**A**–**C**) Screening DIC interference sequences (# P>0.05,* P<0.01,versus Control). (**D**–**F**) Verification of AAVs infection efficiency (# P>0.05,* P<0.01,versus Tg). The data represent as mean ± SEM of a typical series of 3 experiments.

**Figure 6:**
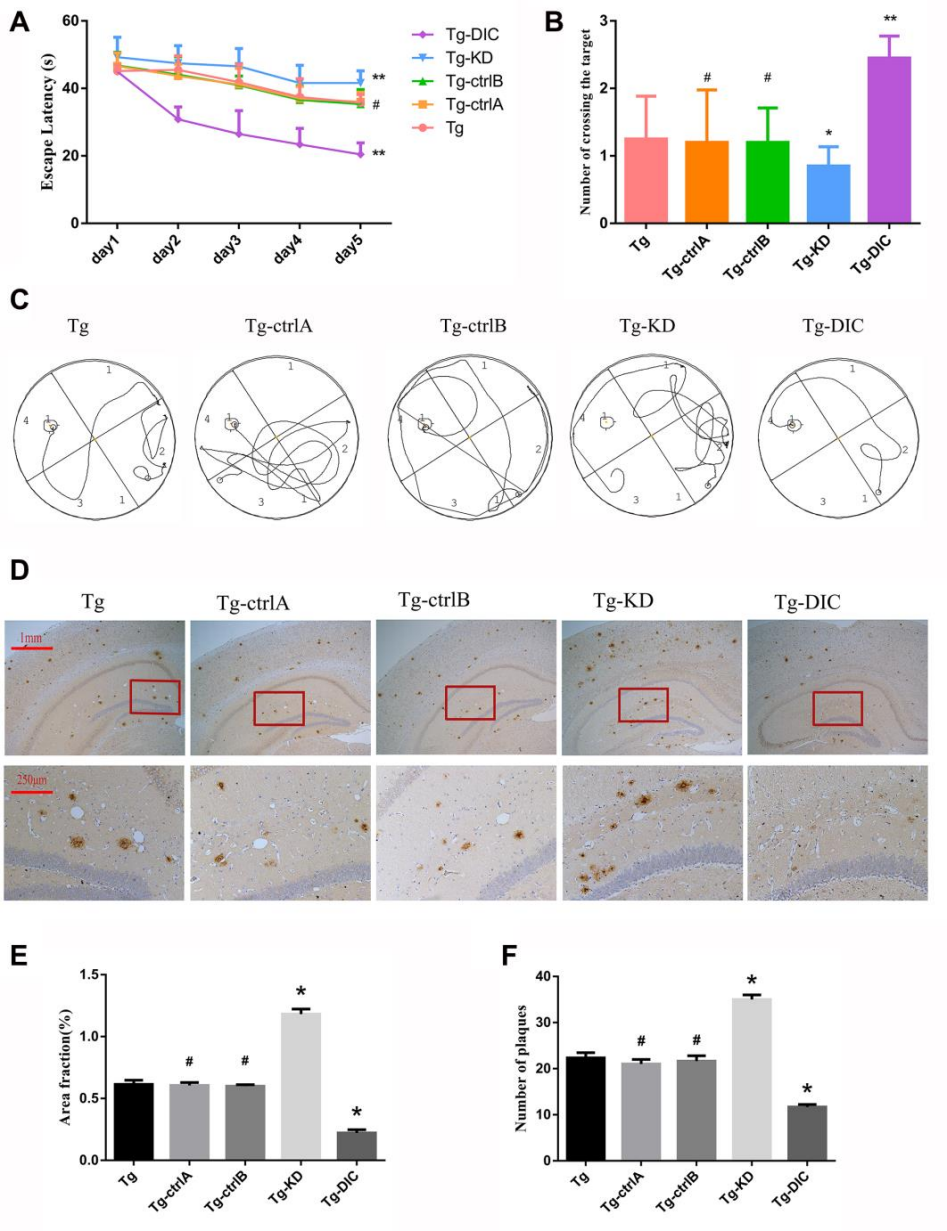
**DIC improved cognitive function and reduced insoluble Aβ deposition in hippocampus of APP/PS1 double transgenic mice.** (**A**) The escape latency during 5 days of the MWM test. (**B**) Number of crossing the target in the last day of the MWM test. (n=5, two-way repeated-measures ANOVA, Tukey's test, # P>0.05,* P<0.05, ** P<0.01,versus Tg.) (**C**) The representative route track map of each group of mice. (**D**) Immunohistochemical staining shows amyloid protein expression in the hippocampus of different groups (Scale bar=1 mm, Scale bar=250 μm). (**E**, **F**) Area fraction and number of plaques of amyloid protein in each group. (# P>0.05,* P<0.01, versus Tg).

Two months after the injection, hippocampus-dependent spatial memory was assessed by the MWM test (representative swim paths for each group are shown in Figure 6C). Compared to Tg mice, Tg-knockdown (Tg-KD) mice demonstrated significantly longer escape latencies (P < 0.05) and DIC-overexpressing Tg (Tg-DIC) mice demonstrated significantly shorter escape latencies (P < 0.05) (Figure 6A), suggesting that DIC knockdown reduces and overexpression improves spatial learning. Further, there were no significant differences among the Tg-ctrlA, Tg-ctrlB, and Tg groups (all P > 0.05). In the probe trial, the number of target area crossings was reduced in the Tg-KD group compared to that in the Tg group, but the difference did not reach significance (P = 0.256), possibly due to a floor effect. However, the number of target crossings was significantly elevated in Tg-DIC mice compared to that in Tg mice (P = 0.002), whereas there were no significant differences among Tg-ctrlA, Tg-ctrlB, and Tg groups (all P > 0.05).

Immunohistochemical staining revealed a greater Aβ amyloid plaque area in the hippocampus of Tg-KD mice (P = 0.000) and significantly reduced area in Tg-DIC mice (P = 0.000) compared to Tg mice (Figure 6D). Alternatively, plaque area did not differ between Tg mice and either Tg-ctrlA or Tg-ctrlB mice (P = 0.723, P = 0.570) (Figure 6E). The number of amyloid plaques in the hippocampus was also significantly greater in Tg-KD mice than in Tg mice (P < 0.001), but it was significantly lower in Tg-DIC mice (P < 0.001). Similar to plaque area, plaque number did not differ between Tg mice and either Tg-ctrlA or Tg-ctrlB mice (P = 0.134, P = 0.433) (Figure 6F).

### DIC promoted the degradation of hippocampal autophagosomes and improved the function of lysosomes in APP/PS1 double transgenic mice

**Figure 7:**
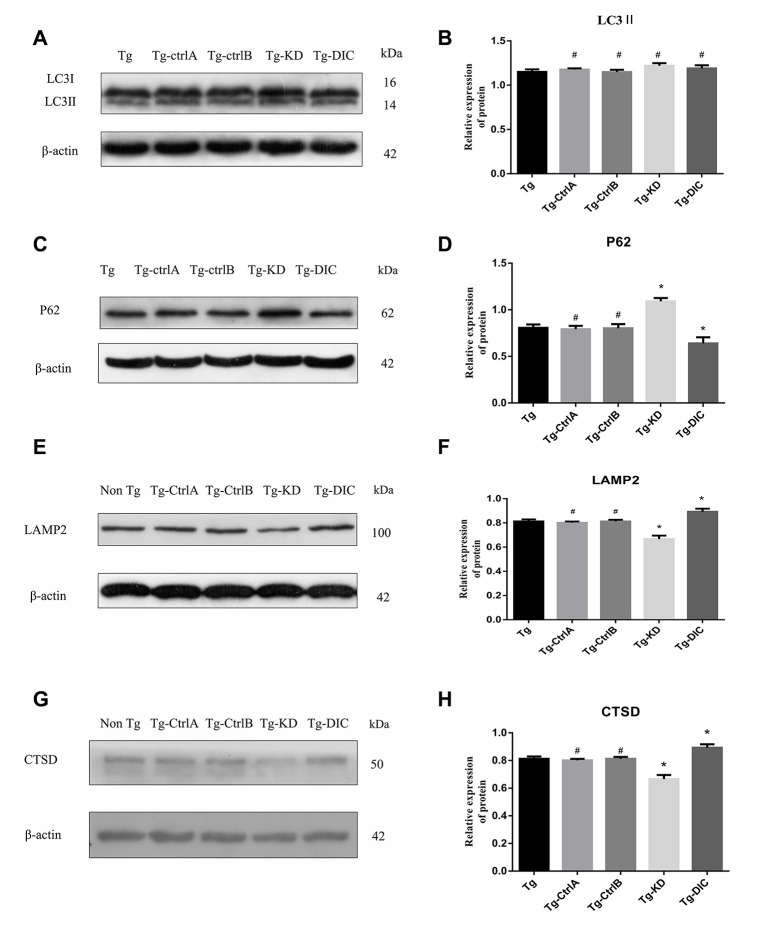
**DIC promoted the degradation of hippocampal autophagosomes and improved the function of lysosomes in APP/PS1 double transgenic mice.** (**A**–**D**) Western blot analysis of (**A**, **B**) LC3II, and (**C**, **D**) P62 in each group. (**E**–**H**) Western blot analysis of (**E**, **F**) LAMP2, and (**G**–**H**) CTSD in each group. The data represent as mean ± SEM of a typical series of 3 experiments. (# P>0.05,* P<0.01, versus Tg).

The expression of LC3II did not differ between Tg mice and the other mouse groups (Tg-ctrlA, Tg-ctrlB, Tg-KD, or Tg-DIC, all P > 0.05) as measured by Western blotting (Figure 7A–7D). Alternatively, P62 expression was significantly higher in Tg-KD mice and significantly lower in Tg-DIC mice than in Tg mice (both P < 0.001), but there was no significant difference in P62 expression between Tg mice and Tg-ctrlA or Tg-ctrlB mice (P = 0.647, P = 0.866).

Compared to Tg mice, Tg-KD mice expressed significantly lower levels of the lysosome marker protein LAMP2 (P < 0.001), whereas Tg-DIC mice expressed significantly higher levels of LAMP2 than Tg mice (P=0.001) (Figure 7G, 7H). There was no significant difference in the expression between Tg mice and either Tg-ctrlA or Tg-ctrlB mice (P = 0.509, P = 0.999). The expression of CTSD was also significantly lower in Tg-KD mice than in Tg mice (P < 0.001), whereas the expression of CTSD was greater in Tg-DIC mice than in Tg mice (P < 0.001) (Figure 7G, 7H). Again, there was no significant difference in CTSD expression between Tg mice and either Tg-ctrlA or Tg-ctrlB mice (P = 0.836, P = 0.742).

### DIC inhibited the expression of KIF5B and enhanced the expression of P150, P50 and Rab7 in hippocampus of APP/PS1 double transgenic mice

**Figure 8:**
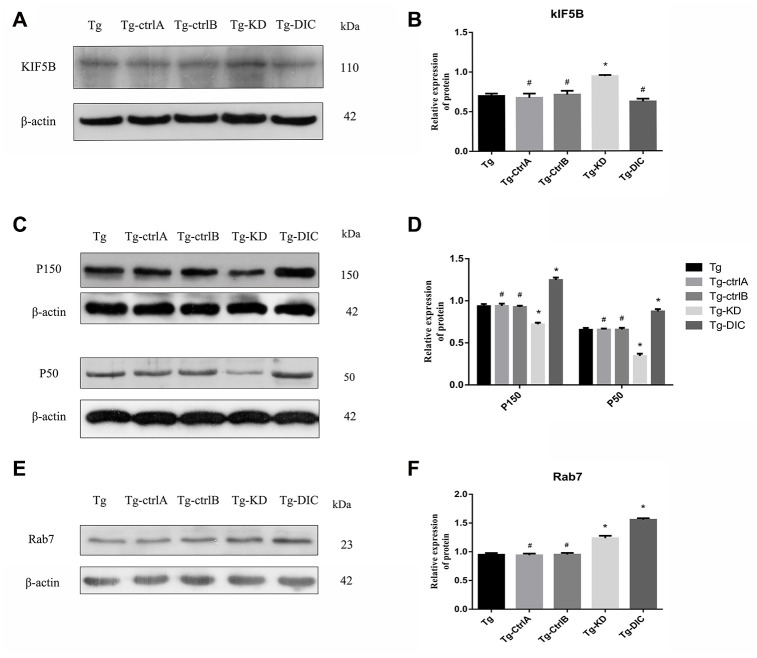
**DIC inhibited the expression of KIF5B and enhanced the expression of P150, P50 and Rab7 in hippocampus of APP/PS1 double transgenic mice.** (**A**–**B**) Western blot analysis of forward axonal transport molecular motor KIF5Bin each group. (**C**–**D**) Western blot analysis of dynactin proteins P150 and P50 in each group. (**E**–**F**) Western blot analysis of Rab7 in each group. The data represent as mean {plus minus} SEM of a typical series of 3 experiments. (# P>0.05,* P<0.01,versus Tg).

The expression of KIF5B was significantly higher in Tg-KD mice (P < 0.001) and lower in Tg-DIC mice compared to that in Tg mice, whereas there were no significant differences among Tg, Tg-ctrlA, and Tg-ctrlB mice (all P > 0.05) (Figure 8A, 8B). Alternatively, the expression levels of P150 and P50 were significantly lower in Tg-KD mice (both P < 0.001) and higher in Tg-DIC mice (both P < 0.000) compared to those in Tg mice, but did not differ among Tg, Tg-ctrlA, and Tg-ctrlB mice (all P > 0.05) (Figure 8C, 8D). Finally, Rab7 expression was enhanced in both Tg-KD mice and Tg-DIC mice compared to Tg mice (P < 0.001), but did not differ in Tg-ctrlA or Tg-ctrlB mice (P = 0.715, P = 0.920) (Figure 8E, 8F).

## DISCUSSION

Protein hydrolysis pathways in neurons gradually weaken with age, resulting in accumulation and deposition of damaged proteins [23]. Both patients with AD and PS1/APP double transgenic mice exhibit large numbers of immature autophagosomes [21, 25], suggesting that protein accumulation in AD may result from disrupted autophagy. Nixon et al. found that the vesicles accumulating in malnourished neuronal processes were actually autophagic vesicles (AVs) containing the intermediate products of the lysosomal pathway [30]. In neurons, axonal transport is critical for full autophagosome maturation and degradation of contents for recycling. Therefore, molecules regulating retrograde axonal transport such as DIC are essential for efficient autophagy, and their deficiency or dysfunction may contribute to aggregation of Aβ, a major pathogenic factor in AD. Indeed, we report that DIC overexpression maintained retrograde transport, autophagy, Aβ clearance, and hippocampus-dependent spatial learning and memory in aging AD model mice.

There are many animal models of AD, of which the APP/PS1 double transgenic mouse model is among the most robust for replicating age-dependent pathogenesis [31]. For instance, these mice show progressively deteriorating performance in the MWM with age [28]. In this study, we demonstrate that this phenotype can be rescued by DIC overexpression and exacerbated by DIC knockdown (Figure 6A–6C). Degraded proteins and damaged organelles accumulate in aging axons due to the polarization of neurons and the limited capacity to upregulate autophagy under stress [10, 32]. Autophagosomes also accumulate in the brain of patients with AD and PS1/APP double transgenic mice [21, 25]. Although autophagy initially increases during the early stage of AD, the transport of autophagosomes and fusion with lysosomes are progressively impaired, which hinders the clearance of cytotoxic Aβ [21, 25]. Therefore, age-dependent failure of autophagy may contribute directly to AD progression.

Amyloid precursor protein (APP) is hydrolyzed by β-secretase and γ-secretase to form Aβ, which can gather as soluble oligomers and insoluble fibers [33]. These aggregating forms can be cleared by autophagy. In this study, the 4G8 antibody was used to detect the 17– 24 amino acid residues of Aβ protein in the brains of Tg and Non-Tg mice. Insoluble Aβ in the hippocampus of APP/PS1 double transgenic mice gradually increased with age (Figure 1D-F) and this occurred in parallel with axonal autophagosome accumulation, indicating reduced autophagic degradation (Figure 2). Overexpression of the retrograde transport effector protein DIC reduced the accumulation of autophagosomes and Aβ deposition in the hippocampus of Tg mice (Figures 6D–6F, 7A–7D), whereas its knockdown had the opposite effects, suggesting that DIC can promote Aβ clearance via autophagy, even during aging in mice prone to Aβ accumulation.

After autophagy is activated in the neurons, autophagosomes created in distal neurites are retrogradely transported to the cell body for fusion with lysosomes, forming autophagic lysosomes [30, 34]. LAMP is essential for lysosome–autophagosome fusion [35, 36]. In AD, Aβ degradation is mediated by several proteases (such as cathepsin), and overexpression of these proteases reduced while their knockdown increased Aβ in mouse brain [37–39]. Mature lysosomes are mainly concentrated in the cell body [40] and maturation is coupled to retrograde axonal transport Retrograde axonal transport of lysosomes is driven by dynein, which demonstrates directional movement at the negative end of the microtubule. The motile active subunits of dynein are located mainly in the protein heavy chain, whereas the direct binding of DIC to snapin mediates the recruitment of dynein to endosomes and lysosomes [14, 41, 42]. In APP/PS1 double transgenic mice, we observed a compensatory increase in the hippocampal LAMP2 expression but decreased CTSD expression, suggesting disrupted association between autophagosomes and the retrograde transport apparatus (Figure 3A–3D). Indeed, overexpression of DIC, which promotes this association, improved lysosomal function in the hippocampus of APP/PS1 double transgenic mice (Figure 7E–7H).

Lysosomes containing hydrolytic enzymes are concentrated around the neuronal cell body or proximal axon, so that endosomes and autophagosomes continuously produced at the distal end of the axon must be transported in the retrograde direction for final degradation of their contents. This process requires specific protein complexes to inhibit anterograde transport of autophagosomes and promote retrograde transport for unidirectional transport along microtubules via dynein–dynactin activity [43]. Tammineni et al. proposed that intracellular Aβ oligomers are associated with the middle and late AVs of neuronal processes in transgenic mice with mutant amyloid precursor protein [44]. Based on the observation of direct DIC binding to Aβ1–42 peptide, it is proposed that excessive intracellular Aβ competitively inhibits snapin-mediated autophagosome recruitment of dynein and thus promotes autophagosome accumulation in distal axons [44]. In our study, the expression levels of DIC and KIF5B were higher in the hippocampus of AD transgenic mice than in Non-Tg mice, but the expression levels of both decreased progressively with age (Figure 3E–3H). The expression of KIF5B was enhanced by DIC knockdown in the hippocampus of APP/PS1 double transgenic mice, whereas DIC overexpression suppressed KIF5B expression, suggesting mutually inhibitory regulation (Figure 8A, 8B).

Dynactin is a multi-subunit protein complex necessary for dynein cytoplasmic activity. The largest dynactin isoform P150 interacts with microtubules and the kinesin complex. In addition, the CC1 and CC2 domains of P150 interact with DIC and other dynactin subunits [45]. Expression levels of both P150 and P50 in the hippocampus were higher in APP/PS1 double transgenic mice than in Non-Tg mice. In Tg mice, dynactin expression may be upregulated due to autophagy or other stressors. However, the expression of both proteins decreased gradually with age, indicating eventual failure of this compensatory mechanism (Figure 4A, 4B). The expression levels of P150 and P50 also decreased significantly under DIC knockdown but increased under DIC overexpression, suggesting that dynein and the helper protein dynactin can act cooperatively to sustain retrograde transport in AD (Figure 8C, 8D).

Autophagosome transport and autophagosome-lysosome maturation are regulated by multiple proteins that bind directly to kinesin and dynein, thereby activating or inhibiting movement [46]. For instance, Rab7 is a vesicular transport-related protein that can promote the fusion of autophagosomes and lysosomes by regulating movement on cell microtubules [12, 13]. Rab7 forms a complex with RILP and ORP1L, and then facilitates dynein–dynactin complex formation, a key factor in autophagosome transport [47]. The expression of Rab7 was elevated in the hippocampus of APP/PS1 double transgenic mice, suggesting a compensatory increase in autophagic flux, but the expression decreased progressively with age (Figure 4C, 4D). However, Rab7 not only recruits dynein to autophagosomes for retrograde transport but also mediates the anterograde transport of autophagosomes from the cell body to axons by facilitating the binding of the regulatory FYVE and coiled-coil protein (FYCO1) to kinesin [48]. Thus, the transport direction of autophagosomes is competitive, but the “molecular switch” regulating the predominant transport direction facilitated by Rab7 remains unknown. The expression of Rab7 did not decrease after inhibition of DIC (Figure 8E), suggesting that Rab7 not only regulates retrograde axonal transport of autophagosomes by binding to dynein but also affects anterograde axonal transport by regulating kinesin. The specific mechanisms require further studies.

**Figure 9:**
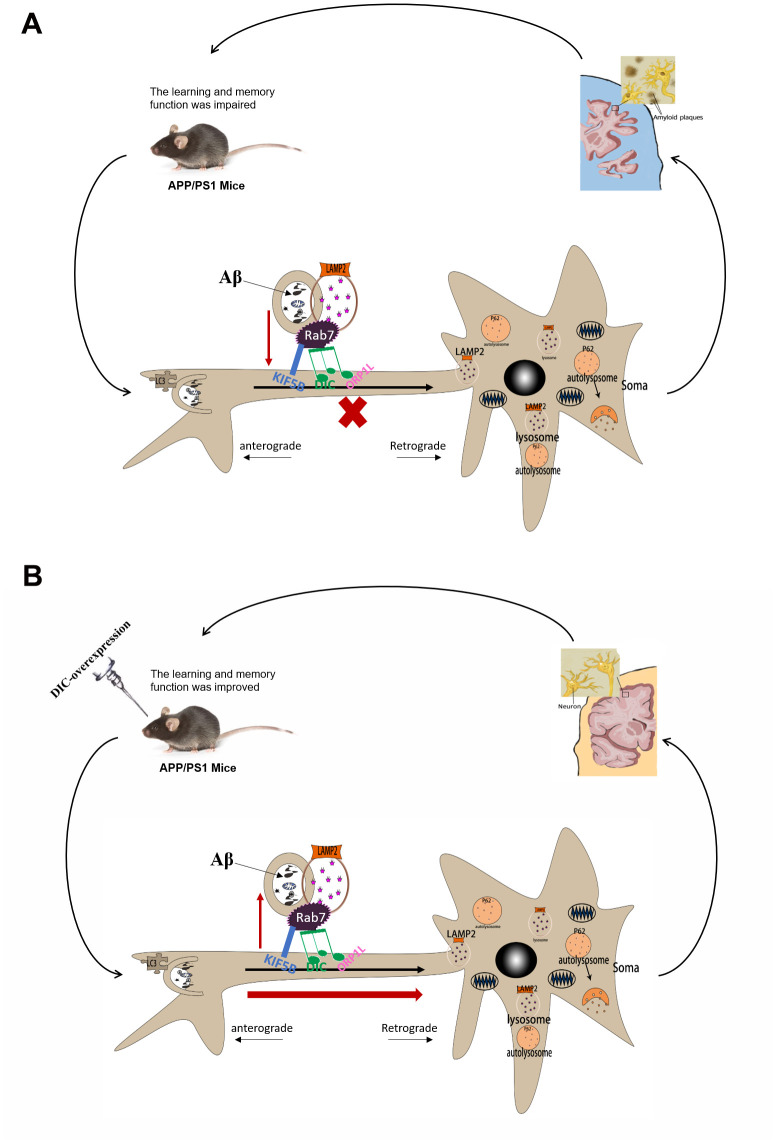
**The mechanism of DIC improves cognitive function by enhancing autophagic retrograde axonal transport-related proteins in AD.** (**A**) Autophagy is activated in the hippocampus of APP/PS1 double transgenic mice. However, the function of lysosome and the process of autophagy axonal transport are impaired, which leads to the accumulation of insoluble Aβ and autophagosomes in the hippocampus. And with the increase of the age of mice, these injuries were gradually aggravated. (**B**) After overexpressing of DIC, the cognitive function of mice was improved and the aggregation of insoluble Aβ decreased. It may be achieved by improving the function of lysosomes and the expression of proteins related to autophagy axonal transport.

In conclusion, autophagy is activated in the hippocampus of young APP/PS1 double transgenic mice but exhibits age-dependent impairment, which leads to the accumulation of insoluble Aβ and autophagosomes (Figure 9A). The overexpression of DIC sustained autophagy, thereby reducing Aβ accumulation and rescuing hippocampal dysfunction (Figure 9B). Therefore, autophagic regulatory proteins are potential targets for AD treatment. One outstanding question warranting further study is why both knockdown and overexpression of DIC enhanced Rab7 expression. Therefore, in future research, we will continue to explore the effects of DIC on autophagic flux and autophagosome axonal transport in vitro to provide new insights and evidence for use in the treatment of AD.

## MATERIALS AND METHODS

### Animals

APPswe/PSEN1dE9 (APP/PSI) transgenic Alzheimer’s disease model mice (C57BL/6J background) and wild-type C57BL/6J mice for breeding were provided by the Institute of Experimental Animals of the Nanjing biomedical research institute of nanjing university. All animals were maintained at 24-26° C on a 12-h light/dark cycle with ad libitum access to food and water. All animal experiments were approved by the Ethics Committee of Animal experiment Center of Chongqing Medical University. All animals used in this study were cared for in strict accordance with the Guide for the Care and Use of Laboratory Animals (NIH Publication No.85-23, revised 1996). All experiments on animals were approved by the Chongqing Medical University Institutional Animal Care and Use Committee (animal certificate number: SCXF2012-0001). 5 per group mice were tested using behavioral experimentation (including Tg mice aged 3 months, 6 months, 9 months and 12 months and Non-Tg mice aged 6 months). Transmission electron microscopy, western blot, and immunohistochemical staining were performed with brain tissue 3 per group mice. More than 20 Tg mice were respectively injected with pAAV-SYN-MCS-EGFP-3FLAG-miR30shRNA(Dync1i2) and pAAV-SYN-Dync1i2-2A-EGFP-3FLAG vector at 6 months of age, composing the Tg-KD group and Tg-DIC group, and age-matched Tg mice were injected with pAOV-SYN-MCS-EGFP-3FLAG and pAAV-SYN-MCS-EGFP-3FLAGcto serve as the two control groups(Tg-CtrlA group and Tg-CtrlB group). In addition, we used 10 age-matched Tg mice without any injection as the Tg group. The behaviors of all mice were examined after injection at 2 months. Western blot, and immunohistochemical staining were performed with brain tissue 3 per group mice.

### Cell culture

Mouse neuroblastoma cell line N2a/WT cells were cultured in the solution of 79% Opti-MEM I Reduced-Serum Medium (opti-MEM, Gibco), 20% fetal bovine serum (Hyclone), 1% solution of penicillin and streptomycin (Beyotime). Cells were maintained in the incubator containing 5% CO2 at 37°C.

### Antibodies and reagents

pAAV-SYN-MCS-EGFP-3FLAG-miR30shRNA(Dync1i2), pAAV-SYN-Dync1i2-2A-EGFP-3FLAG, pAOV-SYN-MCS-EGFP-3FLAG and pAAV-SYN-MCS-EGFP-3FLAG were from Obio Technology (Shanghai, China). The plasmids si-m-Dync1i2_001(CGAAGAGAATGATAGCAAA), si-m-Dync1i2_002(GGTGCTAAGCTGTCATTAA), si-m-Dync1i2_003(GGACAACTAAGAATAACAA), si-m-NC were constructed by Ribo Biotechnology Company(Guangzhou, China). Rabbit anti-β-actin, rabbit anti-LC3B-Specific, rabbit anti- P50, rabbit anti-KIF5B and rabbit anti-P62/SQSTM1 were purchased from Proteintech (US). Mouse anti-P150 was tought from BD. Mouse anti-DIC was bought from Millipore. Mouse anti-Rab7 and rabbit anti-Lamp2 were purchased from Abcam. Mouse anti-4G8(β-Amyloid, 17-24) was bought from Biolegend. All the secondary antibodies were purchased from Bioworld Technology.

### AAV injection

AAVs were bilaterally infused into the 6-month APPswe/PSEN1dE9 (APP/PSI) transgenic mice using stereotaxic surgery (bregma, -2.0 mm; sagittal suture, ±2. 0 mm; depth, -2.0 mm) under isoflurane anesthesia. Then, 1 μl of AAV was infused into each hemisphere with a syringe pump at a flow rate of 0.05μL/min. After allowing 10 min for diffusion into the tissue, the injection needle was withdrawn, the skull was sealed with bone wax, and the wound was closed with surgical staples. Approximately 2 months after injection, brains were extracted and cut into 10-μm frozen coronal sections. Green (excitation wavelength of 493 nm) fluorescence was observed with a Fluorescence Inversion Microscope System of the injection area. A fluorescent signal implied that the labeled gene was expressed and the injection placement was accurate.

### Morris water maze test

The MWM test was used to assess learning and memory capabilities of mice in our study. The apparatus was a white circular pool with a diameter of 100 cm and a height of 50 cm. The pool was imaginarily divided into four equal quadrants that were numbered 1, 2, 3, and 4. The 4^th^ quadrant was the target quadrant with a cylindrical hidden platform (9 cm diameter, 27 cm height) in its center. The pool was filled with water at 23±1°C and was made opaque with nonfat milk powder. Pictures of different shapes, which the mice could use to navigate the maze, are placed on the pool walls of the four quadrants. The experiment includes a visual platform test on the first day and a 5-day hidden platform test in the second to sixth day, as well as a probe trial after the last hidden platform test. In the visible platform test, mice were tested for five contiguous trials with an intertrial interval of 30 min. In the hidden platform tests, mice were trained for six trials with an intertrial interval of 1 h. Mice movement was tracked with a VideoMot2 image analyzer (TSE Systems, China).

### Tissue preparation

All the mice were deeply anesthetized with isoflurane. Some part of mice was perfused with cold 0.9% saline solution. And the brain tissues were preserved in an environment of-80 °C. Some part of mice was perfused with cold 0.9% saline solution and 4% polyformaldehyde (PFA). After that, brain tissues were immobilized in 4% polyformaldehyde (PFA) overnight at 4 °C, dehydrated in 0.1 M phosphate buffer saline (PBS, pH 7.2) in 30% sucrose until they sank and embedded in tissue-embedded media (OCT, SAKURA Tissue-Tek, JP). Coronary hippocampus serial sections (10 microns) were obtained by cryosurgery (CM1860, Leica, GER).

### Transmission electron microscopy

The samples were placed in propylene oxide, embedded in the epoxy resin Epon812, and cut into ultrathin sections. After uranyl acetate and lead citrate double staining, cells were observed by a transmission electron microscopy of Philips EM208S.

### Western blot assay

Hippocampal tissue samples or cells were lysed in cold RIPA pyrolysis buffer(Beyotime, China) and PMSF. Protein concentration was determined by BCA Protein Assay Kit (Beyotime, China) at 570 nm. Equal amounts of protein were loaded in each lane for SDS-PAGE Bis-tris gel and then transferred to polyvinylidene difluoride membranes (Millipore, Billerica, MS, USA). The membranes were washed with blotting buffer (Tris-buffered saline containing 0.1% Tween-20) and then blocked for 120 minutes in the buffer containing 5% non-fat powdered milk. After washed 3 times with blotting buffer, the membrane was incubated at 4°C overnight with primary antibody. After further washing in blotting buffer, the membrane was incubated with secondary antibody at room temperature for 40 minutes. Last, the membranes were developed with ECL Western Blotting Detection Reagents, and Image J was used to quantitate the expression of proteins.

### Immunohistochemistry

The samples were permeabilized with 0.2% Triton X-100 for 10 minutes at room temperature. Normal goat serum was added to the slides and incubated for 30 minutes at room temperature. The staining protocol employed a modified streptavidin-HRP immunohistochemistry procedure (CoWin Century Biotechnology, Inc, China). Briefly, the primary antibody was then added and placed in a humid box overnight at 4°C. The next morning, the slides were washed 3 times with PBST and treated with peroxidase-conjugated streptavidin and visualized by the diaminobenzidine (DAB) Kit (CoWin Century Biotechnology, Inc, China), and sections were counterstained with haematoxylin. Finally, the slides were sealed with neutral gum and observed under Nikon optical microscope.

### RNA isolation and quantitative real-time PCR analysis

Total RNA was extracted from cultured cells using RNAiso Plus (TaKaRa) according to the manufacturer’s instructions. For mRNA expression analysis, the synthesis of cDNA was conducted with 1 ug of total RNA using PrimeSriptTM RT reagent Kit (TaKaRa) and gene expression quantified using SYBR Premix Ex TaqTM II (TaKaRa). All reactions were performed in triplicate. Primer Sequences used: β-actin-F: 5’-CCAAAAGAAGCTGGAA-3’; β-actin-R: 5‘-GGAAATCGTGCGTGACATC-3’; DIC-F: 5‘-CGAATCTTGTTGTTGGAGGTAC-3’; DIC-R: 5‘-AGATGTTACAGCTACTGCCTTT-3’.

### Statistical analysis

All statistical analyses were carried out by SPSS software (version 18.0). The data are expressed as the mean standard error of the mean (SEM). Two-way analysis of variance (ANOVA) was used for statistical comparison, followed by Bonferroni’s post-test with multiple comparisons. Quantitative analysis was carried out with the help of IMAGE software (version 1.44 P, USA). The difference was significant (P < 0.05).
